# The influence of active learning practices on student anxiety in large-enrollment college science classrooms

**DOI:** 10.1186/s40594-018-0123-6

**Published:** 2018-06-12

**Authors:** Katelyn M. Cooper, Virginia R. Downing, Sara E. Brownell

**Affiliations:** Biology Education Research Lab, School of Life Sciences, Arizona State University, PO Box 874501, Tempe, AZ 85287-4501 USA

**Keywords:** Mental health, Anxiety, Anxiousness, Stress, Active learning, Evaluation, Fear of negative evaluation, Clickers, Group work, Cold call, Random call, Science classroom, College classroom, Undergraduates

## Abstract

**Background:**

Over the past decade, the prevalence of anxiety has increased among college-aged students and college counseling centers have become increasingly concerned about the negative impact of anxiety on students. While college in general can be stressful, college science classrooms have the potential to be especially anxiety-inducing because of the sometimes chilly and competitive environment of the class. Further, college science courses are increasingly being transitioned from traditional lecture to active learning where students take an active role in their learning, often through participating in activities such as clicker questions and group work. There is emerging evidence that suggests active learning activities may cause students to feel anxious, but no studies have thoroughly explored why active learning activities in science courses may increase students’ anxiety. Further, no studies have explored whether active learning activities can reduce students’ anxiety. In this exploratory interview study of 52 students enrolled in large-enrollment active learning college science courses, we investigate how three active learning practices, clicker questions, group work, and cold call/random call, increase and decrease students’ anxiety.

**Results:**

Students reported that clicker questions and group work had the potential to both increase and decrease their anxiety. The way the active learning activity is implemented and the extent to which students perceive they benefit from the activity seems to influence the effect of the activity on students’ anxiety. Conversely, students reported that cold call and random call only increased their anxiety. From our interviews, we identified the fear of negative evaluation, or the sense of dread associated with being unfavorably evaluated while participating in a social situation, as the primary construct underlying students’ high levels of anxiety associated with speaking out in front of the whole class when they do not volunteer.

**Conclusion:**

This study illustrates that active learning can both increase and decrease students’ anxiety depending on the way active learning is implemented. We hope that this study encourages instructors to create more inclusive active learning science courses by implementing active learning in ways that minimize students’ anxiety.

## Background

The prevalence of anxiety is increasing among college-aged students, and the negative effect of anxiety on student health and academic performance is a pressing concern for college counseling centers (Center for Collegiate Mental Health, n.d.; Reetz et al. 2014). The American College Health Association, which provides the largest known comprehensive dataset on the health of college students, reported that 60.8% of college students felt overwhelming anxiety within the past year, and 24.2% of students reported that anxiety negatively affected their academic performance (American College Health Association 2017).

### Anxiety in college science

College in general can be anxiety-inducing because many students experience an increase in academic work load coupled with new responsibilities (Misra and McKean 2000; Ross et al. 1999), and science classrooms have been reported to be particularly stressful for some students (Hanson 2009; Koul et al. 2012; Udo et al. 2004). The rigor and difficulty of the subject material of science courses is a common cause of student anxiety (Mallow 2006; Udo et al. 2004). Further, science courses are known to be generally competitive and can foster “chilly,” and even “hostile” environments, which may cause students to experience higher levels of anxiety (Brainard and Carlin 1998; Seymour and Hewitt 1997; Wyer et al. 2001). Students may also feel anxious in science classes because science faculty have been described as “unapproachable” by students (Seymour and Hewitt 1997), and there are often fewer female instructors and instructors of color in science, which has been hypothesized to increase anxiety for students in minority groups (Mallow 2006). Finally, many college science classrooms are large enrollment, particularly at the introductory level, which can elevate student anxiety levels because of the large numbers of students (McKinney et al. 1983).

### Impact of anxiety on college students

High levels of anxiety have been shown to negatively influence student academic experiences in college (McKeachie 1984; Vitasari et al. 2010). More specifically, anxiety has been shown to negatively affect student cognitive and affective outcomes (Bostani et al. 2014; McKeachie 1984; Vitasari et al. 2010). For example, a study on 106 college students enrolled in a general psychology course showed that anxiety can inhibit exam performance if it cannot be resolved in some way (McKeachie 1951). Another study examining anxiety in second year engineering students found that high anxiety led to lower GPAs (Vitasari et al. 2010). Similarly, Culler and Holahan (1980) explored the relationship between anxiety levels and incoming first-semester students’ study habits and performance and found that students with high anxiety had poorer study skills and achieved lower first-semester GPAs than students with low anxiety. Lastly, a recent study found that students with higher general anxiety in biology were more likely to report lower course grades and intent to leave the major than students who report less anxiety (England et al. 2017).

While a high level of anxiety in students has been shown to inhibit student academic performance, in some instances a moderate amount of anxiety has been shown to improve student motivation and performance (Deshpande and Kawane 1982; Jun Zhang 2001; Sarid et al. 2004). The Yerkes-Dodson law (Yerkes and Dodson 1908) suggests that performance increases with mental arousal (stress and anxiety), but only up to a point (Teigen 1994). Once stress is too high, then performance decreases. The relationship between stress and performance can be dependent on the difficulty of the activity (Teigen 1994). For example, if a task is simple or mundane, then students may be unmotivated to complete the task and thus, stress can serve as a useful motivator. Conversely, difficult or unfamiliar tasks require lower levels of arousal in order for students to concentrate, and even moderate levels of stress can negatively impact performance. While some studies exploring the relationship between student anxiety levels and academic performance support the Yerkes-Dodson law, to our knowledge, no studies have been conducted in the context of science, technology, engineering, and mathematics (STEM) (Deshpande and Kawane 1982; Sarid et al. 2004). Additionally, the strength of the relationship between anxiety and performance varies across studies from correlations with extremely negative values to positive values, yet a meta-analysis of 126 published studies exploring the relationship between anxiety and performance yielded an overall negative relationship between anxiety and performance (*r* = − .21) (Seipp 1991). While additional studies and meta-analyses need to be done, the work to date suggests that decreasing high levels of student anxiety, and identifying the factors that increase anxiety could maximize student academic success and retention in science.

### The potentially positive and negative effects of active learning on student anxiety

College science courses are increasingly being transitioned from traditional lecture to active learning because, on average, active learning has been shown to be a more effective way to teach (Freeman et al. 2014; American Association for the Advancement of Science 2015). Active learning is a broad umbrella term to describe courses where students are actively constructing their own knowledge as opposed to listening passively. There are many different ways to enact active learning, but typically active learning includes students working with other students during class and more frequent assessment of student learning in the classroom (Eddy et al. 2015b; Freeman et al. 2014).

Active learning college science classrooms may be particularly anxiety-inducing for students because of the high frequency of situations that could induce a student’s fear of failure. Anxiety has been described as a multifaceted reaction to the threat of failure; the idea of failure can be especially devastating when students put effort into a task because it can imply that they have a low ability or are incompetent, which threatens their self-worth (Covington 1992). College students often fear failure when their academic ability is evaluated (Stipek 1993), which has been termed achievement anxiety (Covington 1992). College students’ academic abilities are commonly evaluated by assessing their performance on a task such as a quiz or exam (Covington 1992; Stipek 1993); these situations are referred to as evaluative situations. Nearly all college science courses have evaluative situations where student achievement anxiety can be activated; the most common evaluative situations in most college courses are exams (Covington 1992). However, active learning courses typically have a greater number of evaluative situations compared to traditional lecture courses because of a larger number of assignments and activities during class. Students can experience achievement anxiety when they evaluate their own learning, such as when a student is working on an in-class assignment and realizes that he or she is unable to solve a problem. Students can also experience achievement anxiety when they are evaluated by other students or the instructor. For example, students may experience achievement anxiety when talking with other students about course content during class if they view that discussion as an evaluative situation where their competence is evaluated by others (Stipek 1993). Similarly, answering a question posed by an instructor can instigate achievement anxiety, especially if the student does not know the correct answer and worries about the instructor’s opinion of them. Thus, because active learning classes are structured with high numbers of activities where students could be evaluated, it is likely that active learning courses have a higher potential to increase student anxiety compared to traditional lecture courses and this could potentially explain why some students are resistant to engage in active-learning.

To our knowledge, no studies have explored students’ anxiety in college active-learning courses across science. However, there is some evidence to suggest that students with high levels of anxiety may struggle in active learning courses more than they do in traditional lecture. A study that explored the experiences of 69 junior college students enrolled in either a teacher-centered section or a student-centered section of an introductory psychology course found that students with high levels of anxiety performed best in the teacher-centered classroom where the instructor discouraged student participation during class, whereas students with low levels of anxiety performed best in the student-centered classroom where the instructor encouraged student participation during class (Dowaliby and Schumer 1973). Similarly, a study in a computer science course found that students with high levels of anxiety performed better in teacher-centered lectures, and students with low levels of anxiety performed better in more cooperative, interactive learning environments (McInerney et al. 1997). While these studies indicate that active learning classes may present challenges for students with high levels of anxiety, they do not explore why students with high levels of anxiety do not perform as well in active learning classes or whether evaluative active learning practices further exacerbate students’ high anxiety. However, a recent study across three large-enrollment biology classes showed that five active learning classroom practices (cold call, volunteering to answer questions, completing worksheets in class, working in groups, and using clickers) all caused students to experience anxiety (England et al. 2017). However, this study only explored the extent to which these active learning practices caused student anxiety exclusively in biology courses and did not explore whether these active learning practices could be implemented or modified in ways to lessen anxiety.

While the study by England et al. (2017) suggests that active learning practices can increase students’ anxiety, there is some evidence to suggest that evaluative active learning practices may also decrease students’ anxiety. For example, one study of pre-service math teachers found that their anxiety was reduced when learning math through “hands-on” approaches (Harper and Daane 1998). Interviews with the preservice teachers found that they enjoyed “doing something” compared to listening to lecture and that a hands-on approach to problem solving helped them better understand math, which caused them to feel less anxious. This study also found that the participants’ anxiety was reduced when they worked in groups to solve math problems. The participants explained that their anxiety decreased because they understood the content better when someone besides the instructor explained it to them and that working in groups allowed them to work through problems in more than one way. In another study that explored anxiety levels of 163 high school students in a science class, the researcher found that students who were randomly assigned to work on science problems in groups, as opposed to working through problems individually, expressed significantly lower anxiety (Okebukola 1986). The author hypothesized that group work reduced students’ anxiety because it helped them to focus their attention on science and made them feel more accepted in the classroom. Thus, while active learning practices may increase students’ anxiety if students fear being evaluated negatively, the same active learning practices may have the potential to decrease students’ anxiety by positively influencing their learning. Yet, to our knowledge, no studies have explicitly explored how active learning practices could have a positive impact on student anxiety. Thus, we aim to explore how and why evaluative situations in active learning courses may increase or decrease student anxiety.

### The current study

In this study, we explore how evaluative active learning practices affect students’ anxiety in the context of large-enrollment science courses. We focused only on evaluative situations that are common in active learning courses; because exams are features of both active learning and traditional lecture courses, we constrained our study and did not include exams. We decided to use an in-depth semi-structured interview approach to explore the nuances of the factors that may influence how active learning practices affect student anxiety in these classes. Individuals have unique levels of enduring anxiety (Turner and Gellman 2013); that is, some people have consistently mild levels of anxiety and others have consistently severe levels of anxiety. In this study, we were interested in exploring how an individual’s standard anxiety level, regardless of how high or low it was, changed as a result of engaging in active learning practices. Specifically, we set out to identify the following:

- What specific aspects of evaluative active learning practices in large-enrollment science courses cause student anxiety to be increased?
- What specific aspects of evaluative active learning practices in large-enrollment science courses cause student anxiety to be decreased?

## Methods

### Interview recruitment

In Fall 2016 and Fall 2017, we administered a short demographic survey to students enrolled in large-enrollment active-learning biology courses (Introductory Biology and Upper-level Physiology) at a research-intensive institution in the southwestern USA. In addition to demographic questions, the survey asked students whether they would be willing to be interviewed about their experience in active learning science courses.

We chose to take a purposive sampling approach (Patton 2002) and recruited students who were enrolled in the large-enrollment active learning biology courses at the end of the semester, so that all students who interviewed had completed at least one active learning science course. Many large-enrollment chemistry courses and some physics courses are also being taught in an active learning way at this institution, so it is likely that students had completed more than one large-enrollment active learning science class at this time. Students were offered a $15 gift card as an incentive to participate in an interview focused on their experience in active learning in science courses. Email recruitments were sent out to 1086 students who had indicated on the demographic survey that they were interested in participating in an interview. Fifty-two students signed up for and came to their interview. The researchers chose to analyze all 52 student interviews and, upon data analysis, were confident that data saturation had been reached, and no additional students were recruited.

### Identifying varying levels of anxiety in students

All students have likely experienced at least mild levels of anxiety as a normal response to stress (Bamber and Schneider 2016), and their anxiety levels may fluctuate depending on life stressors (The National Institute of Mental Health 2016). However, for students with chronically high levels of anxiety, alleviating or exacerbating anxiety within the context of a science classroom could be particularly impactful. Thus, we hoped to interview students with a range of anxiety levels.

To get an estimate of students’ anxiety levels, we asked all interviewees to fill out the Generalized Anxiety Disorder 7-item scale (GAD-7), which measures anxiety on a continuum (Spitzer et al. 2006). The GAD-7 consists of seven Likert-scale questions about symptoms of anxiety with four answer choices ranging from "not at all" to "nearly everyday". We used this measure as an approximation for the extent to which each interviewee experienced high anxiety as an enduring personality trait (Turner and Gellman 2013).

### Interviews

Semi-structured interviews were conducted by two interviewers (K.M.C and V.R.D). We developed a set of interview questions to explore how students’ levels of anxiety were affected in active learning large-enrollment college science courses. After developing the interview questions, we conducted think-aloud interviews to establish cognitive validity of the interview questions with four undergraduate students—two of whom identified as having chronically high anxiety and two of whom did not. The interview protocol was iteratively revised after each think-aloud interview until no questions were unclear or misinterpreted by students (Trenor et al. 2011). During the interview, we defined active learning by referencing the active learning class that the student was recruited from (e.g., “an active learning class such as BIO 101”). We intentionally did not define active learning by referencing common active learning practices such as clicker questions or small group discussions because we did not want to bias student responses by focusing their attention on specific practices. During the interviews, students were asked to describe what aspects, if any, of their large-enrollment active learning college science courses increased their feelings of anxiousness and why. We also asked students what aspects, if any, decreased their feelings of anxiousness and why. The semi-structured nature of the interviews allowed us to explore interesting topics that emerged in an interview with one student that may not have emerged in every interview. Interviews were audio-recorded and transcribed upon completion. The average interview time was 45 min, and interviews ranged from 30 to 60 min. Data were anonymized, and pseudonyms have been given to each of the students. We suspected that students had not previously been asked about how their experience in large-enrollment active learning science courses might influence their anxiety, so we gave students a handout with some of the interview questions just before the interview began and allowed them ~ 5 min to write down their thoughts about each question. We have previously found that this helps students give more complete answers to interview questions, particularly when the subject that is being explored is stigmatized (Cooper and Brownell 2016). Students were told that they could use the piece of paper as a reference during the interview.

### Interview analysis

Two researchers (K.M.C. and V.R.D.) reviewed every interview and identified the most prominent active learning practices that were mentioned by students when asked what specific aspects of large-enrollment active-learning science classes influence their feelings of anxiousness. The two researchers identified three practices—clicker questions, group work, and cold call/random call—that were mentioned by at least 50% of students (26 students) during the interviews. We did not ask students specifically about any of these practices, yet these practices emerged from the interviews. We chose to exclusively explore these three active learning practices that emerged in the majority of interviews to maximize the chance that we had interviewed enough students to reach data saturation for each active learning practice. Two researchers (K.M.C. and V.R.D.) independently reviewed half of the interviews (26 interviews each). The researchers separately analyzed each interview transcript for what aspects of each active learning practice—clicker questions, group work, and cold call/random call—increased and/or decreased students’ anxiety. For each active learning practice, the researchers allowed themes to emerge from the data and took notes throughout the analysis and reconvened to discuss their findings using constant comparative methods. Specifically, the researchers used their notes to develop themes and then discussed what quotes from the interviews they reviewed fell under which themes. This constant comparison of quotes was meant to ensure that the description of the theme adequately represented all quotes within the same group and that the quotes were not different enough from one another to warrant a separate theme (Glesne and Peshkin 1992). The researchers determined that there were no themes that were unexplored and that data saturation had been reached within the current sample and no further recruitment was needed (Guest et al. 2006). Together, the researchers developed a coding rubric for what elements of each active learning activity influenced student anxiety levels. The researchers then individually coded all 52 interviews using the coding rubric and then compared their codes. The reviewers came to consensus about any portion of an interview that they had coded differently.

This study was approved by the university’s institutional review board.

## Results and discussion

We present the results and discussion together to help elaborate on our findings and contextualize them with previous literature. We also do not report out specific percentages of students who perceived a practice to increase or decrease their anxiety because we did not ask every student explicitly about how each practice influenced their anxiety and instead allowed students to bring up a particular practice that increased or decreased their anxiety. We did not identify any trends about whether students with differing anxiety levels (minimal, mild, moderate, or severe) were more or less likely to report that a specific practice increased or decreased their anxiety. However, we did note that students with minimal anxiety were less likely to mention any of the three active learning practices that we explored in their interview than students with higher levels of anxiety. We present how each active learning practice affected each of the 52 students that we interviewed in Table 1.

**Table 1:** Student demographics and report of how active learning practices influence each student’s anxiety

	Student demographics						Whether a student reported that an active learning practice increased their anxiety, decreased their anxiety, or did not affect their anxiety		
Pseudonym	GAD score (0–21)	General anxiety level based on GAD score	Class	Gender	Race/ethnicity	First-generation college going	Clicker questions	Group work	Cold call/random call
Viviane	2	Minimal	Intro bio	Female	Asian/Pacific Islander	No	X	X	–
Felicia	2	Minimal	Upper bio	Female	Hispanic/Latino	Yes	–	↑ ↓	↑
Marcus	3	Minimal	Upper bio	Male	Black or African American	No	–	–	–
Jessica	3	Minimal	Intro bio	Female	White	No	↓	–	–
Dawn	3	Minimal	Intro bio	Female	Asian/Pacific Islander	No	–	↓	–
Bill	4	Minimal	Upper bio	Male	White	No	–	↓	–
Xavier	4	Minimal	Upper bio	Male	Black or African American	Yes	–	–	↑
Sally	4	Minimal	Upper bio	Female	White	No	↑	–	↑
Rodger	4	Minimal	Upper bio	Male	White	No	↓	↑ ↓	↑
Kathryn	5	Mild	Upper bio	Female	White	No	–	↑	–
Craig	5	Mild	Upper bio	Male	White	No	X	↑	↑
Taylor	5	Mild	Intro bio	Female	White	No	↑ ↓	↓	↑
Evan	5	Mild	Upper bio	Male	White	No	X	↓	↑
Lisa	6	Mild	Upper bio	Female	White	No	X	–	–
Parker	6	Mild	Upper bio	Other	White	No	↑	↑	↑
Giselle	6	Mild	Upper bio	Female	Hispanic/Latino/a	Yes	↑	↑	↑
Rachelle	6	Mild	Upper bio	Female	Black or African American	Yes	↑	↑	↑
Shannon	6	Mild	Intro bio	Female	White	No	–	X	↑
Shawna	6	Mild	Intro bio	Female	Asian/Pacific Islander	No	–	X	↑
Claire	6	Mild	Upper bio	Female	Hispanic/Latino/a	No	X	X	↑
Kenna	6	Mild	Upper bio	Female	White	No	X	X	↑
Rick	7	Mild	Upper bio	Male	White	No	↑ ↓	–	–
Mya	7	Mild	Intro bio	Female	Black or African American	No	–	–	↑
Jordan	7	Mild	Upper bio	Female	White	No	–	↑	↑
Megan	8	Mild	Intro bio	Female	White	No	↑	↑	–
Gloria	10	Moderate	Intro bio	Female	Hispanic/Latino/a	Yes	↑	↓	–
Carter	10	Moderate	Upper bio	Male	Black or African American	No	↓	↓	–
Tiffany	11	Moderate	Upper bio	Female	White	No	↑	↑	–
Anita	11	Moderate	Upper bio	Female	Hispanic/Latino/a	No	X	↑	–
Charlotte	11	Moderate	Intro bio	Female	Hispanic/Latino/a	No	–	↑	↑
Olivia	11	Moderate	Upper bio	Female	White	No	↑	↓	↑
Theodore	12	Moderate	Upper bio	Male	White	No	–	↑	–
Quinn	12	Moderate	Upper bio	Female	Hispanic/Latino/a	No	↑ ↓	↑ ↓	↑
Serena	12	Moderate	Upper bio	Female	Hispanic/Latino/a	Yes	–	↓	↑
Lidia	13	Moderate	Intro bio	Female	Hispanic/Latino/a	No	↑ ↓	↓	↑
Antoinette	14	Moderate	Intro bio	Female	White	No	↑ ↓	↓	–
Lindsay	14	Moderate	Intro bio	Female	White	No	↑	–	↑
Blanca	14	Moderate	Upper bio	Female	Hispanic/Latino/a	Yes	–	↑	↑
Celeste	14	Moderate	Upper bio	Female	Asian/Pacific Islander	Yes	↓	↓	↑
Cindy	14	Moderate	Upper bio	Female	White	No	↓	↓	↑
Kit	15	Severe	Intro bio	Female	White	Yes	↓	↓	–
Emmy	15	Severe	Intro bio	Female	Hispanic/Latino/a	No	↑	–	↑
Brittany	15	Severe	Intro bio	Female	White	No	–	↑	↑
Alana	15	Severe	Intro bio	Female	Hispanic/Latino/a	No	↑	↑	↑
Kristen	15	Severe	Intro bio	Female	White	No	–	X	X
Iris	17	Severe	Upper bio	Female	Hispanic/Latino/a	Yes	↑	↑	–
Paige	17	Severe	Upper bio	Female	White	No	–	↓	↑
Anne	20	Severe	Upper bio	Female	White	Yes	↑	↓	–
Cole	20	Severe	Upper bio	Male	Hispanic/Latino/a	Yes	↑	↑	↑
Morgan	21	Severe	Upper bio	Female	White	No	↑	↑ ↓	–
Monya	21	Severe	Upper bio	Female	Hispanic/Latino/a	Yes	–	↑ ↓	–
Owen	NA	NA	Upper bio	Male	White	Yes	–	–	↑

↑ indicates that a student highlighted a specific element of an active learning practice that increases their anxiety, ↓ indicates that a student highlighted a specific element of an active learning practice that decreases their anxiety, X indicates that a student reported that a specific active learning practice does not influence their anxiety levels, and – indicates that the student never mentioned the specific active learning practice during their interview

### Student population

A demographic profile of each student is reported in Table 1. Of the students interviewed, 76.9% identified as female, 21.2% identified as male and one student identified as non-binary. The majority of students (53.8%) identified as White, 28.8% identified as Hispanic, Latino/a, or Spanish, 9.6% identified as Black or African American, and 7.7% identified as Asian or as a Pacific Islander. Seventy-three percent of students identified as a continuing generation college student, and 26.9% of students identified as a first-generation college student. Students’ experience with anxiety varied across our sample. We used the GAD-7 scoring rubric to classify students’ level of generalized anxiety (Spitzer et al. 2006). Seventeen percent of students reported minimal generalized anxiety (GAD-7 score < 5), 30.8% of students reported mild generalized anxiety (GAD-7 score 5–9), 28.8% of students reported moderate generalized anxiety (GAD-7 score 10–14), 21.2% reported severe generalized anxiety (GAD-7 score ≥ 15), and one student was unwilling to complete the Generalized Anxiety Disorder Scale.

### Description of recruitment courses

Students were recruited from active learning biology classes: Introductory Biology and Upper-level Physiology. The instructors of Introductory Biology and Upper-level Physiology reported, and students confirmed, that they regularly used clicker questions during class and asked students to work with at least one other person during nearly every class period. In Introductory Biology, the instructors would sometimes call on students who did not volunteer to answer a question in front of the entire class, but they did not formally implement random call where a student is randomly selected from a list to answer a question in front of the whole class. In Upper-level Physiology, the instructors never called on students to speak out in front of the class. However, in addition to the classes that we recruited from, students also reported experiencing clicker questions, group work, and random call in other active-learning science courses that they had been previously or were currently enrolled in. For example, many of the students who were enrolled in the Upper-level Physiology reported that they were concurrently enrolled in a genetics course where the instructor regularly practiced random call.

### Active learning practice no. 1: the influence of clicker questions on student anxiety

Clicker questions are often used by instructors as a way to improve student conceptual understanding and to gather immediate feedback from students during class. Instructors typically pose multiple-choice clicker questions to all students during class and students answer anonymously using personal response devices or clickers. Instructors are usually able to immediately interpret the frequency of correct student responses, which instructors can use to inform and adjust their teaching in real time (Sun et al. 2014). Because each student has a registered clicker, instructors can also use clickers to promote accountability in class, ranging from giving students participation points for “clicking in” to awarding points only for correct responses.

Using clicker questions during class has been championed as an active learning activity that allows instructors to collect feedback from individual students anonymously and simultaneously, which prevents students from changing their answers to conform to academically higher status students, which can happen when students raise their hand to indicate an answer (Kennedy and Cutts 2005; Stowell and Nelson 2007; Sun et al. 2014). Further, clicker questions have been shown to encourage an increase in student engagement (Bode et al. 2009; Dallaire 2011; Stowell and Nelson 2007; Sun et al. 2014; Trees and Jackson 2007) and improve academic performance (Anthis 2011; Elicker and McConnell 2011; Kennedy and Cutts 2005; Stowell and Nelson 2007).

During the interviews, 26 students (50.0%) indicated that clickers influenced their anxiety in some way. We identified specific ways in which clicker questions affect student anxiety, which are summarized in Table 2.

**Table 2:** Factors that influence student anxiety about clicker questions

	Timing	Grading		Understanding of science concepts		Understanding how science knowledge compares to others	
	Student does not have enough time	Points awarded for completion	Points awarded for accuracy	Student confirms what concepts they do/do not understand	Student clarifies understanding	Student realizes other students struggle with concepts	Student realizes they know less than other students
Effect on student anxiety	↑	↓	↑	↓	↓	↓	↑

↑ indicates a factor that increases student anxiety; ↓ indicates a factor that decreases student anxiety

#### Timing and grading of clicker questions

Students identified that their anxiety in science classrooms increased when they felt they did not have enough time to think through a clicker question. For example, Lindsay explained that she feels as if she is a “slow thinker” and can feel rushed during clicker questions, which exacerbates her anxiety.

Lindsay: “Clicker questions are stressful (…) I'm a very slow thinker. I don't know what is wrong with me, but I'm a very, very slow thinker. I'm rushed into things. Being rushed causes me anxiety.”

Other students, such as Taylor and Megan, highlighted that clicker questions were particularly anxiety-inducing if they did not feel as though they had enough time to think through the question and if points were awarded for correct answers. Students explained that if they did not have enough time to fully engage with the question, it was likely that they would get the question wrong. If getting the question wrong meant that they also lost points, their anxiety was further exacerbated.

Taylor: “If [clicker questions] are timed, it causes me to feel anxious. Because [when the question is for points], it’s like I need to get something in, and I’m going to get it wrong.”

Megan: “Clicker questions make me anxious when they’re timed. In one of my science classes, my professor would time the clicker questions and the amount of time we have to click in. If there was any material that you had to calculate or something, I didn't always do it in time. I would lose my whole points for that day even though I was in the class and I was present.”

Megan went onto describe a positive feedback loop where her increased level of anxiety influenced her ability to think through the clicker question, which in turn further exacerbated her anxiety. She explained that she becomes focused on getting the points as opposed to focusing on learning the content.

Megan: “When I feel anxious, it’s almost that I can't solve the problem or answer the question clear-mindedly because I'm so scattered and worried about getting my answer in on time (…) I can't think clearly so if I were to click in a question or have an answer, I don't know if my answer was the correct answer because I'm so worried about getting my points that day that I feel that I don't know. I’m not always having the clearest mind.”

Students suggested that, when using clickers, instructors could provide points for participating as opposed to points for accuracy, which would reduce their anxiety. However, some studies show that students are likely to learn more, as measured by getting the correct answer more frequently, if instructors reward correct answers with points, likely because it increases student accountability (James 2006; Willoughby and Gustafson 2009). Yet, as Megan’s quote suggests, penalizing students who give incorrect answers by not awarding points may encourage students to focus their attention on points instead of focusing their attention on learning. In a study exploring student anxiety in college math, the authors found that students who have goals associated with the desire to achieve favorable grades (performance oriented) are more likely to experience anxiety than students who are most interested in learning and mastering the material (learning-goal oriented) (Ironsmith et al. 2003). Thus, it is possible that by timing clicker questions, instructors are inadvertently shifting students to adopt more performance-oriented attitudes, which may heighten their anxiety. However, we propose that there are ways to implement clicker questions that may reduce anxiety while still increasing student accountability. For example, instructors can pose a clicker question to students and have them answer individually. Then, the instructor can allow students to discuss with their neighbors and answer again. If the instructor grades the first attempt on participation and then the second attempt on accuracy, it improves students’ chances that they will get the question correct (Smith et al. 2009) and also allows them to think through the question the first time without the pressure of getting the question correct. However, the instructor would need to make this grading explicit to students or else students may assume that every question is graded on accuracy. We will further discuss the potential benefits of allowing students to work together on clicker questions in the finding about the relationship between student anxiety and group work.

#### Understanding of science concepts and comparing knowledge with others during clicker questions

We also found that clicker questions can affect student perceptions of their own learning, which influences their anxiety. Students explained that instructors’ use of clicker questions helped them clarify concepts and deepen their understanding of the presented material, which is consistent with prior literature encouraging instructors to integrate clicker questions into the classroom (Knight et al. 2013; Smith et al. 2009; Smith et al. 2011). Being provided with an opportunity to strengthen their understanding of science seemed to reduce many students’ feelings of anxiety. For example, Kit explained how clicker questions help her feel as though she has a more complete understanding of the material, which she perceived to reduce her anxiety.

Kit: “If anything, I feel like the active learning part reduces my anxiety (...) I feel like I have a more complete understanding of the material (…) The clicker questions really helped me feel like I’m getting a more complete understanding of [the material].”

Students also explained that even if a clicker question did not help them understand a concept, simply being able to identify what concepts they do and do not understand seemed to lessen their anxiety because then they knew what to focus on when studying. For example, when comparing traditional lecture and active learning science courses, both Celeste and Rodger describe that active learning activities such as clicker questions allow them to identify what they do or do not understand, whereas they do not have the same opportunity to check their understanding in traditional lecture courses.

Celeste: “The active learning, you know what you know by clicker questions, by answering questions, so you know what you understand and what you don't understand. In traditional lecture courses, you're just given the material and [the instructor says] ‘I'll see you during the test, let's see what you get wrong or right.’”

Rodger: “In the passive learning, in traditional lecture, the anxiety levels are pretty high. It's sort of like a plateau, you kind of plateau at this really high level [of anxiety] because you're trying to jot down information in a notebook for an hour, and then there's no clicker questions or there are no assignments and stuff like that, so you don't know if you actually know that information.”

For most students, getting a single clicker question incorrect did not seem to exacerbate their anxiety unless they felt as though they were one of a few students out of the whole class who did not understand the concept. For example, Lindsay and Parker describe what it feels like to be in the minority group of students who get a question incorrect.

Lindsay: “I feel anxious when I feel like I am in the wrong science class. For example, when everyone else understands [the concept], and I don’t. When [the instructor] puts up that graph [after a clicker questions] and says ‘All these people say C, and this majority says D,’ or something. I'm usually the B people. In that moment, I'm like, ‘How are people understanding it?’ I feel so dumb. I don't understand how people get it, and I can't.”

Parker: “If I really tried on the question and really don’t understand the concept and see that on a graph, 90% of the class knows this and I’m in the 10% that got the question wrong. I guess I’m not doing great. Then especially for me, with my anxiety, it can really affect me.”

Although displaying a histogram that shows 95% of the class got a question right may be a way to highlight the success of most of the class, we do not know of literature that supports that this is beneficial for students who got the question right. However, these student interviews suggest that showing that graph may increase anxiety for students who answered the question incorrectly. Based on these interviews, we would recommend instructors not show a histogram when all but a few students selected the correct answer. Alternatively, if an instructor prefers to show the histogram, they may want to consider practicing error framing, or framing students’ mistakes or misconceptions as natural or useful (Bell and Kozlowski 2008). An instructor can practice error framing by explicitly telling the class that it is OK to answer clicker questions incorrectly, by explaining that an incorrect answer is a common misconception, or by suggesting that he or she understands why students might think an incorrect answer was correct. Error framing has been shown to decrease student anxiety about making mistakes (Bell and Kozlowski 2008), increase student motivation (Steele-Johnson and Kalinoski 2014), and improve students’ connections with faculty (Cooper et al. 2018a).

In conclusion, most of the student-described anxiety about clicker questions could be classified as achievement anxiety because students’ anxiety seemed to stem from a fear of losing points or realizing that they are underperforming compared with other students in the class. However, clicker questions were identified as a way to decrease a broader level of achievement anxiety that related to the students’ achievement in science because clicker questions helped them to identify what science topics they do and do not understand, as well as helped them to deepen their knowledge about particular subjects in science.

### Active learning practice no. 2: the relationship between group work and student anxiety

Group work is commonly integrated into active learning classrooms because student collaboration to achieve learning goals has been shown to improve student attitudes toward science and increase student achievement (Johnson and Johnson 2009; Johnson et al. 2014; Springer et al. 1999; Tanner et al. 2003). Further, group work allows students to hear and provide diverse opinions as they work toward solving science problems (Cooper et al. 2017a; Lamm et al. 2012). Instructors can integrate group work at any point during an active-learning class. For example, students can work with each other during clicker questions, while engaging with a worksheet, or when the instructor presents an open-ended problem to the whole class. Thus, we chose to explore how working with others affects students’ anxiety levels, independent of what activity the group is working on.

Of the students whom were interviewed, 36 students (69.2%) indicated that group work affected their anxiety in some way. Table 3 highlights the specific aspects of group work that influence students’ anxiety.

**Table 3:** Factors that influence students’ anxiety about group work

	Relationship with working partners		Understanding of science concepts		Understanding how science knowledge compares to others	
	Student is comfortable with work partner	Student is uncomfortable with work partner	Student confirms what concepts they do/do not understand	Student clarifies understanding	Student realizes other students struggle with concepts	Student realizes they know less than other students
Effect on student anxiety	↓	↑	↓	↓	↓	↑

↑ indicates a factor that increases student anxiety; ↓ indicates a factor that decreases student anxiety

#### Relationship with peers in group work

In this study, we found that much of student anxiety in active learning stems from a fear of being evaluated negatively and in the case of group work, students fear being evaluated negatively by a peer or group of their peers. The fear of being negatively evaluated while participating in a social situation such as group work, or even while simply anticipating participating in group work, is termed fear of negative evaluation (Watson and Friend 1969; Weeks et al. 2005). Fear of negative evaluation was described by many of the students in the study, such as Megan, Craig, and Alana, when they talked about how group work influenced their anxiety.

Megan: “If I were talking in a small group and I was not knowledgeable on the topic of the question, then I would feel anxious [because] I would feel more judged by somebody just because I don’t want to feel or sound stupid that I don’t know what I’m talking about.”

Craig: “If I realize that I answered a question wrong when talking with people in my group, it makes my anxiety a little worse. I’m sitting there thinking ‘Oh man, the person next to me probably thinks I’m dumb because I just shared with him the wrong idea.’”

Alana: “I feel less anxious in traditional [lecture] class because there’s not that social aspect involved (…) In active learning I worry, ‘What are [other students] going to think of me? They probably think I’m dumb for not knowing [the answer to a science question].’”

While many students’ fear of negative evaluation seemed to have a negative impact on their experience in the class, this was not true for all students. For example, Theodore described that working with other students caused him a little anxiety when he does not know something but it seemed to motivate him to study more, which has been described as one of the benefits of moderate anxiety levels and why some instructors may perceive that anxiety can be beneficial for students (Jun Zhang 2001).

Theodore: “It is a little bit anxiety causing when you don't know something, but the person next to you does. There's a little bit of a disconnect in the conversation because one person obviously knows a lot more or maybe did the reading when the other person didn't. That happened to me a couple of times, so it was like, ‘This person is way more ahead than I am’ (…) It kind of indicates that maybe I should've read more or something.”

The difference in students’ responses to fear of negative evaluation may be partially explained by the Yerkes-Dodson law; a low fear of negative evaluation, as described by Theodore, may motivate students to study more, while a more severe fear of negative evaluation may have a negative effect on students like Blanca and Parker, whose fear of negative evaluation can be so severe that it can cause them to think about the experience even after they have left their science class.

Blanca: “I've spent up to a week thinking about [what I’ve said to my groupmate] (…) I embarrass myself, then I think about it the next time I see them, I'm like, ‘What if they bring up last time, that I didn't know the answer? Or what if they make a joke?’ Some of the people like to make jokes about, ‘Remember last time?’ And then I just want to avoid the situation.”

Parker: “[My anxiety during group work] goes back to the central theme of being judged. Some things I’ll say will keep me awake at night. It’s like, ‘Did I overshare? Did I not talk enough?’”

Many students, including Cindy, described that their fear of negative evaluation was sufficiently decreased if they had developed a positive relationship with other students in their group because then they perceive that they are less likely to be judged by that person.

Cindy: “I feel less uncomfortable bouncing ideas off of [my friend in class] because, I guess when you say something to someone and it's the first thing you've ever said to them, it's like a big impact. It makes a big impression, or it feels that way. Whereas, [my friend] has known me for a year, so I feel like even if I say something stupid she still knows that I'm smart.”

Rodger echoes Cindy’s experience; he describes that he is more likely to share ideas if he feels comfortable with the person he is working with. He elaborates on the benefits of feeling comfortable sharing more ideas, which he perceives allows him to think more creatively and develop more unconventional ideas.

Rodger: “I think a lot of the anxiety in classes comes from the people around you- trying to find someone that you're comfortable with or can talk to. You feel more comfortable being around that person and sharing ideas. And, if we were trying to work through a problem in class, maybe [I’m more likely to] throw out more unconventional ideas. [If you’re talking with someone you don’t know] it's like, ‘Shoot, I should've said [my idea] because it would've been cool if I got [the question] right.’ So being able to share those ideas with people around you and get their feedback (…), I think that's huge for having more creative thinking and thinking outside of the box.”

To maximize student comfort, reduce anxiety, and maximize idea sharing, instructors could consider allowing students to choose which other students they work with during class. Previous research suggests that allowing students to choose their own groups during active learning could be anxiety-reducing for students and particularly beneficial for female and LGBTQIA students (Cooper and Brownell 2016; Eddy et al. 2015a; Theobald et al. 2017). However, this current study suggests that allowing students to choose whom they work with may reduce all students’ anxiety because they can choose partners who they are most comfortable with.

If instructors decide that they want to assign groups, we suggest allowing students to have sufficient time at the beginning of class to introduce themselves and try to quickly establish a level of comfort with each other. Asking students to use name tents, or to write their name on a piece of cardstock that they bring to class, may be another way to allow students to build more personal relationships, especially in large-enrollment courses; students have reported that name tents help them get to know students around them and build community in the classroom (Cooper et al. 2017b). Additionally, explicitly talking with students about the importance of sharing ideas, even wrong ideas, and stressing how important it is to let all students share their ideas, may alleviate some students’ anxiety and encourage more equitable participation (Cooper et al. 2017a). Being forthright with students about the importance of equitable participation may also help students without anxiety realize that a student may be quiet in a group, not because they do not have anything to say, but because they are afraid of how others might react (Cooper et al. 2017a). Lastly, allowing students’ time to think or write before asking them to share their thoughts could also help alleviate anxiety about group work because students have time to synthesize what they would want to contribute to the discussion (Tanner 2013).

#### Understanding of science concepts and comparing knowledge with others during group work

Students also recognized group work as a way to enhance their knowledge about science, which decreased their anxiety because they perceived that it maximized their ability to do well in the course, on exams, or more broadly in science. Specifically, students, like Quinn, valued that group work allowed them to recognize what science content they do and do not know.

Quinn: “Discussing [science] really helps [decrease my anxiety] because once you get the input of other people, even if you are wrong, it does change your answer and you're like ‘I can see how they got there or why they got there.’ (…) Even if my friend and I are both confident about our different ideas, we can be like ‘let’s go through them and see what’s wrong.’ Then you feel extra good because you’re like ‘I can recognize what I don’t know and what I do know.’”

Other students, such as Felicia and Antoinette, highlighted that group work helps them learn more than when they are only listening to the instructor lecture.

Felicia: “I loved discussing [science] with my classmates. Not only do you learn [science] from the professor, you're learning it from your classmates in different terms. So the professor might be not saying it in ‘English,’ but your classmate might say it in ‘English,’ so for sure, definitely the active learning style was so helpful in decreasing my anxiety.”

Antoinette: “There is anxiety when I am like ‘Shoot. I don’t know what the professor was talking about.’ I get more anxious. [My anxiety] comes down when I talk to the students around me. Then we talk about the concept, then I'm like, ‘Okay. I understand this. It's not that hard.’ (…) I really like group work, to be honest, just because we all just teach each other, that helps a lot.”

Felicia and Antoinette’s shared opinion that it can be beneficial to hear science described by their classmates in different terms than the instructor uses is supported by previous research that shows that students recognize the benefit of learning science from other students who think more like novices and less like the expert instructor (Chi et al. 2004; Cooper et al. 2018b; Harper and Daane 1998). Further, studies have shown that peer instruction, or asking students to explain concepts to each other during class, improves student performance on formative assessments such as clicker questions (Crouch and Mazur 2001; Smith et al. 2009).

We also found that allowing students to work with each other caused them to evaluate their own understanding as it compares with other students’ understanding. A student’s perception of their intelligence as it compares with other students’ intelligence in a specific domain, such as physiology or biochemistry, has been defined as academic self-concept (Brunner et al. 2009; Marsh and Craven 1997; Shavelson et al. 1976). Previous work from our research group shows that female students are particularly prone to having low academic self-concept; that is, compared with males, females are more likely to perceive that they are less smart than their groupmate, even when they have an equivalent or higher GPA than their groupmate (Cooper et al. 2018c). In this current study of student anxiety, we found that students frequently compared their understanding with others during group work. For some students, such as Monya, comparing themselves with their classmates exacerbated their anxiety if they perceived that they are less smart than the student or students whom they were working with.

Monya: “I usually have friends in my classes with me and they usually know more than I do, so I'm just constantly freaking out and thinking that I don't know as much as they do.”

Conversely, we found that if a student interacts with someone in their group and realizes that they both struggle with material, it seemed to reduce their anxiety, as illustrated by Anne.

Anne: “When I’m have having trouble with [content] it’s like ‘It’s probably because I'm stupid and don't understand.’ But then [talking with other students helps me realize] ‘OK, everyone else is struggling with the same concept.’ Or, ‘Someone else has the same question.’ So I think it probably helps with more dense concepts and subjects that are just complicated. It kinda helps students relate to other students as well.”

Because achievement anxiety has been conceptualized as an ability-linked reaction to failure (Covington 1992), research suggests that achievement anxiety can be decreased when ability-protecting excuses are available to students (Covington 1981). For students in this study, recognizing that their groupmates also struggled with science content seemed to serve as an ability-protecting excuse; that is, when students realized that other students also struggle with science, they were less likely to perceive their own ability as low, which meant that their perception of their own academic ability was “protected,” consequently decreasing their anxiety.

In conclusion, students described that working with other students during class could induce achievement anxiety if they feared that students in their group would view them as low achieving or incompetent. Conversely, students reported that group work could decrease their anxiety because it helps them learn and helps them to realize that other students also find science challenging.

### Active learning practice no. 3: the negative effects of cold call/random call on student anxiety

Instructors asking students to share their thoughts in front of the whole class without them volunteering to do so is a popular form of formative assessment and has been suggested as an evidence-based active-learning strategy to increase student participation in class (Dallimore et al. 2013) and student learning (Eddy et al. 2015b). There are two common ways that this is done: cold call and random call. Cold call is when instructors either call students by name or point to students to answer a question when the student has not volunteered to answer the question. Random call is a form of cold call, where an instructor randomly calls on students to answer a question in front of the whole class, using a randomly generated list of student names.

Students in this study overwhelmingly reported that when instructors practiced cold call or random call in large-enrollment science courses, it only increased their anxiety and never decreased their anxiety. Thirty-two students (61.5%) mentioned cold call during their interview. Thirty-one students (59.6%) reported that cold call or random call increased their anxiety and one student (1.9%) said it did not affect their anxiety. In contrast to the other active learning practices explored in this study, not a single student mentioned that cold call or random call could decrease their anxiety.

#### Fear of negative evaluation underlies student anxiety during cold call/random call

Students’ anxiety about instructors practicing cold call or random call seemed to be driven by the fear of negative evaluation or the sense of dread associated with the potential to be negatively evaluated by others (Watson and Friend 1969), as illustrated by Celeste and Lidia.

Celeste: “That’s what I’m afraid of when getting called on in front of the whole class, getting it completely wrong, or not saying anything. (...) If you don't know, you're that one person who seems stupid. That's what I feel like. Not knowing the answer makes me feel anxious, makes me feel like I’m the outcast, the stupid one.”

Lidia: “Having to speak in front of a large group of people makes me anxious. It’s the fear of being wrong or sounding dumb- being embarrassed.”

Some students acknowledged that their fear of being evaluated was exacerbated when cold call was practiced in large classes.

Parker: “Smaller class sizes [compared to large classes] would decrease my anxiousness. It's like a comfort thing, less people looking at me. Logically, I know that people don't actually do this, but it's fewer people who are going to be like ‘That kid in class said something stupid today.’ It's fewer people who are going to be potentially talking about me. I know logically that's not something that I should be worried about, but [it still increases] anxiety.”

While students seemed to recognize that instructors likely practice cold call or random call to enhance their learning, they felt as though the anxiety associated with the anticipation of speaking out in front of others negatively impacted their learning and performance. More specifically, students, such as Celeste, Quinn, and Emmy, described that they were unable to think through a science problem posed to the class because they were afraid of being called on by the instructor.

Celeste: “My brain stops. [If the instructor] asks me a question, I have no idea what the answer is. If you were asking me in a small group, yes I'll tell you the answer and get it. If in a large group, my brain just stops. I have no idea why.”

Quinn: “I think it's the pressure of not only having to answer a question in front of a professor who clearly knows the answer, but in front of all your peers as well [that causes me anxiety]. It kind of clouds your thinking and then you feel like you can't think at all and it just gets worse. Your heart is racing, you start to sweat, and your brain just shuts off. You're looking for an answer, but then you can feel that there's pressure there so you're not actually thinking of a good answer.”

Emmy: “It also happened once in chemistry, [the instructor] just pulled me up and he's like, ‘Hey. You're going to answer this question.’ For me if it's unexpected, I really don't like it. I just forget everything. I don't know what I'm doing. Then I'm really nervous because I feel like everyone is watching you. I don't know what to do. (…) I freeze up and I can't really say the answer but I kind of have to have something come out in order for the teacher to be happy.”

These students’ experiences are consistent with literature that suggests that individuals with fear of negative evaluation focus a significant amount of their attention on monitoring their environment for a possible threat of evaluation, such as the threat of being called on in front of the whole class, and therefore, have less cognitive capacity to engage in other activities, such as thinking through a science problem (Heimberg et al. 2010). Additionally, anxiety has been hypothesized to be debilitating for a student’s performance when a task (e.g., speaking in front of the class) is introduced in a way so that poor performance can reflect negatively on the student (Sarason 1973; Stipek 1993).

Students, such as Serena, Jordan, and Emmy, also reported that when they were called on and had to speak out in front of others, their anxiety caused them to lose their train of thought or they were unable to clearly articulate their ideas, which in turn further heightened their anxiety.

Serena: “Being randomly called on, that level of anxiety, it just throws me. [When anticipating being called on by the instructor] I knew the answer in my head, but just being in that moment [being called on], I just wasn't able to put those thoughts into a clear, coherent sentence. It just made me feel bad. I felt sick to my stomach. It doesn't really help you because then you're just inhibited.”

Jordan: “I have the thought in my head, but it doesn’t come out necessarily the way I want it to. It’s hard to explain myself.”

Emmy: “I’ll spit something out and the teacher is like, ‘I don’t understand. Can you restate that?’ I’ll have to sit there and figure out how to reword what I just said without sounding like an idiot.”

High levels of anxiety have been predicted to be especially detrimental to learning when students are required to hold and manipulate speech-based information (Eysenck and Calvo 1992; Owens et al. 2008; Rapee and Barlow 1991) and can prevent students from clearly articulating their thoughts in front of others. Further, if students experience fear of negative evaluation, they are likely evaluating their own behavior (e.g., monitoring if they are sweating, misspeaking, and stuttering), which increases cognitive load and can compromise their ability to successfully articulate their thoughts about science in front of hundreds of other students (Heimberg et al. 2010).

While a previous study suggests that practicing cold call or random call should cause students to become more comfortable speaking out in class (Dallimore et al. 2013), we found that cold call and random call had the opposite effect on the students who we interviewed. Many students described that if they experienced being called on, especially if they struggled to think through the science question or articulate their thoughts, they were less likely to want to participate in the future.

Emmy: “[After being called on in front of the class] I sit there and I’m like ‘I can’t believe I didn’t know the answer to that.’ I beat myself up over it. I’m still really nervous about it afterwards because it’s like, ‘I can’t believe that happened.’ (…) Then, I become a lot more quiet. I don’t put myself out there anymore. It kind of hits a switch and I’m not going to participate as much in class after that point. I feel like I don’t want to do that again. (…) Usually when those anxiety attacks are that extreme, I try to stay away from anything that’s going to promote the same thing.”

Quinn: “[When instructors cold call students] it really just puts me more on edge because I know that feeling and I know [getting called on randomly] could happen again. It's not like one of those things you’re like ‘Oh it's over, it wasn't that bad’. No, it was that bad, and I did not like it. A lot of people stop coming to lecture for that very reason, which I’m sure is the opposite of what [the instructor] wanted.”

Students wanting to avoid cold call or random call after they have had a negative experience, or even after they perceived that a classmate has had a negative experience, is consistent with psychology literature which suggests that if students have a negative experience in class, then their fear of negative evaluation, and consequently their anxiety, is only going to be exacerbated in future situations (Heimberg et al. 2010). Thus, we would predict that repeatedly exposing students to cold call or random call would not help to decrease their anxiety unless they had a positive experience, which none of the students in our study described (Heimberg et al. 2010).

Random call has been recommended as an alternative to hearing out from volunteers in class as a way to create a more equitable classroom environment (Eddy et al. 2014; Tanner 2013) because certain students can dominate whole class discussions, either because they are more willing to volunteer to share an answer or because instructors are more likely to call on particular students (Eddy et al. 2014). However, evidence suggests that, compared to their male peers, females experience disproportionately more anxiety than males in whole class discussions (Eddy et al. 2015a), which suggests that while cold call and random call may be more equitable with regard to which student voices are heard, they may not afford students an equitable experience during class because females may still experience more anxiety than males in those whole class discussions. Further, despite the calls to hear out from students in front of the whole class, we can find little evidence that directly links cold call or random call in science courses to student benefits such as student-learning gains. In a recent study by Broeckelman-Post et al. (2016), students in a large-enrollment college biology course reported that the practice of cold calling students during class encouraged them to pay attention, attend class, discuss ideas, and listen to other students. Yet, these same students also reported experiencing anxiety as a result of cold call, and students with anxiety disorders reported that the practice of cold calling resulted in frequent absences from class, and heightened their anxiety and a “sense of feeling under pressure when in class” (Broeckelman-Post et al. 2016, p.31). We only found the negative effects of cold call in this study: the students whom were interviewed in our current study suggested that they ultimately struggled to pay attention in class because once the threat of cold call or random call was introduced, they were preoccupied with worrying about how others might perceive their intellectual capability if they were to be called on. Given the degree to which cold call can increase student anxiety and lack of evidence for the benefits of cold call, it seems as though we as a community could find alternative ways to engage students in class that would not elicit high anxiety for students.

Considering the literature on the negative effects of high anxiety on student learning and performance and the results of this study, in addition to the paucity of evidence on the benefit of cold call or random call on student learning in science, we suggest that instructors consider other means of sharing student ideas with the whole class. While we do not recommend only hearing out from students who volunteer because this has been shown to lead to gender inequities in whose voices are heard (Eddy et al. 2014), we suggest an alternative where instructors can walk around during group work and gather ideas from students and then share those ideas out with the entire class. This allows instructors to transform students’ ideas into complete, accurate thoughts before reporting them out, which can reduce anxiety for the student who would have shared and can also lead to less confusion for other students in the class who may not have understood the response shared out by the student. Furthermore, if instructors are thoughtful about sharing out ideas from students whose identities are underrepresented in science, this practice may even be a way to promote equity in the classroom. We acknowledge that many variables contribute to how instructors facilitate whole class discussions, including the size and layout of the classroom, so we do not assume that there is one solution that works for every classroom.

In conclusion, we found that cold call and random call only exacerbated students’ anxiety and no students identified ways in which these practices could decrease their achievement anxiety. Overwhelmingly, students’ anxiety seemed to be rooted in a fear of negative evaluation.

### Is there a benefit to increasing student anxiety?

As the Yerkes-Dodson law suggests, moderate levels of anxiety may enhance students’ performance in active-learning classes, especially if the task is simple or mundane (Yerkes and Dodson 1908; Teigen 1994). Thus, increasing students’ anxiety may be a way to motivate students to attend class, complete a worksheet, or read the textbook. However, as shown by students’ GAD scores, students’ general levels of anxiety can be remarkably different and increasing students’ anxiety may differentially impact students because of their different levels of generalized anxiety. Therefore, if an instructor’s goal is to increase student motivation, especially with regard to a cognitively difficult task, then it is important to consider how increasing anxiety may differentially affect students, especially students, such as females and lower-performing students, who are known to have higher levels of anxiety (Kessler et al. 2005; McKeachie 1984; The National Institute of Mental Health 2016). More research needs to be done to better understand how moderate levels of anxiety affect student performance. Because high levels of anxiety have been shown to negatively impact student performance and retention in STEM (Teigen 1994; England et al. 2017), we suggest that, if instructors are interested in increasing student motivation, it may be worthwhile to consider altering other factors that have been shown to enhance student motivation without negatively impacting performance and retention such as increasing instructor immediacy (Allen et al. 2006; Baker 2010; Cooper et al. 2017b), building students’ academic self-concept (Ommundsen et al. 2005), or helping students see the relevance of what they are working on (Theobald et al. 2015).

### Limitations

We acknowledge that this study was done in the context of one institution. Although students were asked about their experiences in science classrooms, students were only recruited from introductory biology and upper-level physiology courses, and we did not differentiate whether a student was talking about a specific type of science class (biology, chemistry, physics) when answering a question. Before broad generalizations can be made, a more systematic analysis of these practices with larger numbers of students should be done. Further, we only report here on three active learning practices. There may be other active learning practices that generate greater levels of anxiety that we did not explore. Finally, because this study was exploratory and we did not ask every student about whether a specific practice increased or decreased their anxiety, we did not systematically analyze whether there were differences in how active learning practices affected students with minimal, mild, moderate, and severe anxiety. However, this would be an important area of future research.

## Conclusions

High levels of anxiety have been shown to inhibit students’ academic performance and persistence in science (Teigen 1994; England et al. 2017). This study aimed to explore the relationship between active-learning practices and student anxiety in hopes of providing instructors with information about how to minimize students’ high-anxiety in their classrooms. The three active learning practices that were explored in this study, clicker questions, group work, and cold call/random call, all had the potential to increase students’ anxiety. Fear of negative evaluation was identified as a construct underlying students’ achievement anxiety during active learning activities. Both clicker questions and group work also had the potential to decrease student anxiety because students felt that these active learning practices helped them to learn science. We identified specific aspects of clicker questions, group work, and cold call/random call that can negatively impact students’ anxiety levels and we hope these findings will help instructors to create more inclusive active learning science classrooms.

## Abbreviations

STEM: Science, technology, engineering, and mathematics
